# Evaluating Varied Label Designs for Use with Medical Devices: Optimized Labels Outperform Existing Labels in the Correct Selection of Devices and Time to Select

**DOI:** 10.1371/journal.pone.0165002

**Published:** 2016-11-08

**Authors:** Laura Bix, Do Chan Seo, Moslem Ladoni, Eric Brunk, Mark W. Becker

**Affiliations:** 1 Michigan State University School of Packaging; East, Lansing, Michigan, United States of America; 2 Cognitive and Cognitive Neurosciences Group, Department of Psychology, Michigan State University; East Lansing, Michigan, United States of America; 3 Statistical Consulting Center, CANR Biometry Group, Michigan State University; East Lansing, Michigan, United States of America; University of California Irvine, UNITED STATES

## Abstract

**Purpose:**

Effective standardization of medical device labels requires objective study of varied designs. Insufficient empirical evidence exists regarding how practitioners utilize and view labeling.

**Objective:**

Measure the effect of graphic elements (boxing information, grouping information, symbol use and color-coding) to optimize a label for comparison with those typical of commercial medical devices.

**Design:**

Participants viewed 54 trials on a computer screen. Trials were comprised of two labels that were identical with regard to graphics, but differed in one aspect of information (e.g., one had latex, the other did not). Participants were instructed to select the label along a given criteria (e.g., latex containing) as quickly as possible. Dependent variables were binary (correct selection) and continuous (time to correct selection).

**Participants:**

Eighty-nine healthcare professionals were recruited at Association of Surgical Technologists (AST) conferences, and using a targeted e-mail of AST members.

**Results:**

Symbol presence, color coding and grouping critical pieces of information all significantly improved selection rates and sped time to correct selection (α = 0.05). Conversely, when critical information was graphically boxed, probability of correct selection and time to selection were impaired (α = 0.05). Subsequently, responses from trials containing optimal treatments (color coded, critical information grouped with symbols) were compared to two labels created based on a review of those commercially available. Optimal labels yielded a significant positive benefit regarding the probability of correct choice ((P<0.0001) LSM; UCL, LCL: 97.3%; 98.4%, 95.5%)), as compared to the two labels we created based on commercial designs (92.0%; 94.7%, 87.9% and 89.8%; 93.0%, 85.3%) and time to selection.

**Conclusions:**

Our study provides data regarding design factors, namely: color coding, symbol use and grouping of critical information that can be used to significantly enhance the performance of medical device labels.

## Introduction

At the turn of the millennium a flurry of publications illuminating a high prevalence of preventable patient injuries resulting from medical care catalyzed an international movement focused on increased patient safety [1–4].

Although drugs are a mainstay of Western healthcare and have been a central focus of the patient safety movement, medical technology and devices are assuming an increasingly prominent role in healthcare [5–7], and it has been suggested that understanding the “role of devices in medical harms and hazards has lagged behind advances in other areas of safety concerns such as medications.” [8]

Regardless of whether caused by medication or medical device, incidents resulting in adverse events are generally multifactorial in nature, with latent factors, faults, errors and mistakes aligning to enable an event’s occurrence. Lack of clarity and/or confusion associated with label layout and information is one of many factors with potential to contribute to the likelihood of an adverse event.

Medical device labeling provides information that is targeted to a care giver or patient that is associated with a given device, and, for many devices, the labeling is an essential component of safe and effective use [9]. General labeling requirements for medical devices that are sold in the US can be found in Chapter 21 of the Code of Federal Regulations (CFR) part 801. At minimum, device manufacturers must prominently label:

- The name and place of the business (21CFR 801.1)
- The intended use of the device (21 CFR 801.4) and
- Adequate directions for use (21 CFR 801.5)

Beyond this, there are labeling requirements that are related to the specifics of the devices themselves, such as those containing latex (21CFR 801.437) or that are delivered in a sterile state.

The communication of information about medical devices, and the use of labeling to do so, has been referred to as “increasingly important,” but it has been indicated that there is “currently insufficient empirical evidence on how practitioners utilize and view labeling.” [10] What is known in this regard has not directly measured effects, but instead been exploratory and qualitative in nature, primarily employing focus groups [10, 11] and surveys [10] to assess optimal labeling practices and identify problems with existing labels.

Stifano et al. [10] conducted a series of nine focus groups in three cities with 77 healthcare practitioners for the purpose of identifying the information on medical device labels that was effective at drawing attention and satisfying provider needs. Practitioners suggested the need for concise, non-technical information with clear graphical depictions. Results suggested several pieces of information as critical, specifically: instructions for use, warnings, precautions, contraindications, troubleshooting, manufacturer’s contact information, device name, serial number and expiration date.

Findings were echoed in focus groups conducted with operating room personnel by Cai [11] who’s objective was to identify the most prevalent issues associated with medical device packaging. Two major problems emerged as critical: the labeling of medical devices and difficulties associated with sterile transfer. A central theme regarding labeling that was identified through content analysis was that non-critical information hindered healthcare personnel from finding critical information easily. Four pieces of information emerged as critical: expiration dating, sterility status, latex status and product name.

Seo [12] provided further support of these findings through a benchmarking study which characterized the labeling of 20 indwelling, urinary catheters comprising six manufacturers. Placement of the four pieces of information indicated by Cai [11] as critical to providers was enumerated by dividing the catheter labels into quadrants and identifying the location of critical information. Not a single catheter in the study had all four pieces of information in the same quadrant; 40% of those characterized used 2 of the four quadrants to display the four pieces of information and a majority (60%) used three locations to display the information.

Varied recommendations have been made in the effort to improve the labeling of medical devices. Cai’s [11] study participants suggested that critical information should facilitate quick identification via formatting that is easily read without significant interference from non-critical text. One controversial recommendation that emerged as a consistent theme was the use of color-coded information; some participants consistently recommended its use, while others felt that it resulted in significant potential for confusion. DeVenio [13] suggested that medical device labeling would be made more accessible by incorporating “visual language- a method to communicate information primarily using illustrations and graphics instead of words” due to the fact that visual elements (i.e. symbols) can be interpreted regardless of literacy level or language barriers.

The US Food and Drug Administration (FDA) is interested in promoting symbol use as a means of communicating information on the labeling of medical devices. They have recently passed a regulation that allows the use of symbols without adjacent, explanatory text present on the label [14] (provided that the symbol definition is present in an accompanying glossary). This harmonizes US regulations with EU Directives, which encourage the use of symbols without text so that information can be conveyed to speakers of varied languages in limited amounts of space [15].

Herein, we fill an important, identified gap in knowledge [10] by objectively measuring the effect that varied graphic elements (namely, grouping information, color coding information, boxing information and using symbols) have on the correct selection of a medical device (select the latex free product, select the sterile product, select the expired product) and time to correct selection.

## Materials and Methods

All methods were conducted in accordance with those approved by the Biomedical and Health Institution Review Board (BIRB) at Michigan State University #13–698 using a written consent process that required each subject to sign an approved consent form prior to testing.

### Subjects

Eighty-nine healthcare professionals were recruited at the Association of Surgical Technologists (AST) conferences in Savannah, GA and Denver, CO, and using a targeted e-mail of AST members within a 30 mile radius of Lansing, MI. Respondents (primarily surgical technologists and nurses) filled out a survey that encompassed demographic information and information about their work history and work environment prior to viewing 54 test trials on a computer screen.

### Materials

**Fig 1:**
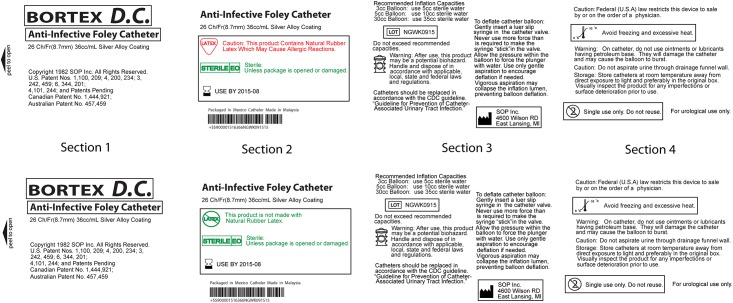
Example of two labels shown in a trial (symbol present, color coded, information grouped, box present). Sections for the two labels are “yoked” so that the same section appears in the same area of each of the two labels (top and bottom). Users are asked to identify the product containing latex as quickly as possible by pressing ↑ for the top label or ↓ for the bottom label. Each of the labels (top and bottom) are presented at the same level of treatment with regard to color, grouping, symbol use or boxing of critical information.

Novel labels depicting information typically present on indwelling, urinary catheters were developed using Adobe Illustrator CS 3.0. Each label image measured 1280 pixels x 768 pixels, and was subdivided into four label sections that were roughly equivalent. This subdivision of sections enabled positional randomization of panels, or “information quadrants” during our test trials. These randomizations were “yoked” such that the sections, and therefore the two comparative labels (top and bottom), were the same in all aspects other than the difference involving the critical information relating to the selection criteria in the pair ((e.g. latex containing vs latex free) (Fig 1)).

Labels were created to allow us to evaluate four graphic design factors, each at two levels: boxing of critical information (boxed and unboxed), grouping of critical information (grouped and ungrouped), symbols representing critical information (absent or present) and color coding of critical information (absent or present). Conditions were crossed for a total of sixteen treatments of interest (2 x 2 x 2 x 2).

**Fig 2:**
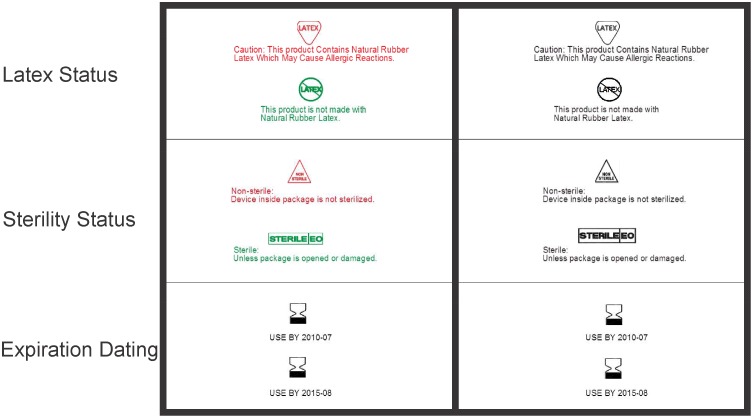
Color Coding for Critical Information. In order to compare our labels which purposefully tested varied factors of graphic design with existing commercial labels, two label designs were created based upon a benchmarking study that we conducted with 20 commercial labels from six different brands (Fig 3-depicting a trial with one of the two designs developed based on commercial labels).

The color coding scheme that was developed carefully considered the feedback of Cai’s [11] focus groups that suggests that systems lacking standardization and/or poorly designed systems have the potential to result in confusion and error. As such, we developed a simple system of color coding that only employed two colors (green, signaling less risk; and red; signaling caution). Information regarding sterile products was listed in green, while their non-sterile counterparts were provided in red. Similarly, products that were indicated to contain latex presented this information in red, while their latex free counterparts provided the information in green for those treatments where color applicable (Fig 2).

### Procedure

During each trial, two labels appeared on the screen, both of which incorporated the same design features (Fig 1). These labels differed in one aspect, a single piece of one of the three pieces of critical information was changed (e.g. one label represented a product containing latex while the other did not). Forty-eight trials were conducted with mock brands (16 treatments (2 x 2 x 2 x 2) x 3 pieces of information) and six trials comprised of mock brands which emulated commercial products based on our benchmarking results (3 pieces of critical information x 2 brands). As such, each subject completed 54 trials in total. Testing was conducted using E-Prime 2.0 (Psychology Software Tools, Inc.), and trial order was randomized to mitigate any run order effects. As previously discussed, images were divided into 4 sections, with individual sections yoked and randomized to mitigate any location effect.

Just prior to each trial, participants viewed an instruction screen that directed them to select a product as quickly as possible in a choice that involved one of the three pieces of critical information (i.e. select the sterile device; select the latex containing device or select the expired device). Selection was made by depressing either “↑” (UP ARROW) or “↓” (DOWN ARROW) on a keyboard entry system (corresponding with the label in the upper or lower position, respectively) within 1 minute.

The position of the correct choice was counter-balanced between subjects for each combination of treatments. For instance, if the latex containing product for a label that had color, grouping, symbols and boxed information appeared on top for subject one, it would appear on the bottom for subject two. For each subject, a correct choice for half of the trials (27) occurred in the top location and the half in the bottom. This was accomplished with two sets of stimuli, A & B, which were rotated between subjects; trials within set were randomly ordered. Forty-four subjects participated in the type-A forced choice task; 45 subjects, in the type-B forced choice task.

### Data Collection

Two response variables were collected for analysis using the E Prime^®^ software for each forced choice task trial:

- A binary variable: Correct choice (Yes/No) prior to timing out at 60 seconds; in the event that a subject timed out (i.e. they didn’t identify a product within 60 seconds), the result was analyzed as a no regarding the correct choice
- A continuous variable: Time taken to make a correct choice (milliseconds) prior to timing out at 60 seconds.

## Results

### Subject Demographics

Of the eighty-nine healthcare professionals recruited for this study, 16 were male and 73 were female. The majority of participants were employed in an acute care hospital (41.6%) or in a college setting as an instructor of nursing students or surgical technologists (37.1%). The remaining were spread among ambulatory/day surgery (6.7%), nursing home/long term (1.1%), public/community health (2.2%), student (7.9%), and other (3.4%). The average age of participants was 45 years old (SD 10.6 years). On average, the subject population reported an average of 21 years of experience in healthcare.

### Data Collection of Responses

A generalized linear mixed model was fitted to the binary variable—correct choice (yes/no, or timeout at 60 seconds) using a logit-link function to model the probability of correct choice (in %) for the 48 trials that involved our mock labels; commercial label trials were not included in this data analysis. Linear predictors in this model were four design factors (grouping, boxing, symbol use and color coding), and all possible 2-way, 3-way and 4-way interactions. From the demographic information collected, ethnicity (p = 0.0230) was retained in the final model, based on their Type III p values (α = 0.05). The model was fitted using the GLIMMIX procedure of SAS 9.3 (SAS Ins., Cary, NC). Estimated least square means (LSM) and corresponding 95% confidence intervals (CI) were reported in the percentage of probability of correct choices. 4,053 (94.9%) out of the 4,272 trials (48 trials with the design elements of interest x 89 subjects) resulted in correct choices; 219 trials (5.1%) had the incorrect product chosen.

There was evidence of significant effects of two main factors involving design variables significantly impacted the probability of correct choice: boxing of critical information (p = 0.0101) and symbol use (p<0.0001). Surprisingly, treatments where data was boxed had poorer rates of performance than trials where critical information was not boxed; specifically, mean rate of a correct response was 95.7% (CI 92.5, 97.5) when the critical information was displayed inside a box compared with 97.1% (CI 94.9, 98.4) for trials without the critical information boxed. Conversely, the presence of symbols significantly aided users in the correct selection of products. The presence of symbols resulted in a significantly higher rate of correct choices than when labels did not have symbols present; specifically, treatments where symbols were present resulted in mean probability of 97.7% (CI 96.0, 98.8) relating to the correct selection of a product compared to a 94.4% (CI 90.6, 96.8) rate for treatments where symbols were absent.

**Fig 3:**
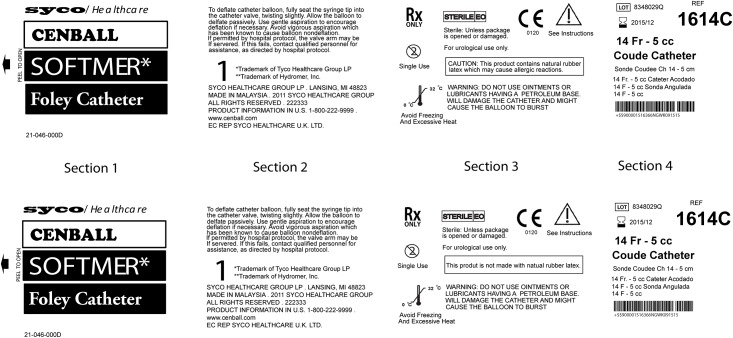
Trial comprised of a Commercial Label. For the sake of comparison, two commercial labels (one depicted here as it would appear in a single trial) were created based on the results of a benchmarking we conducted with 20 labels for indwelling, urinary catheters comprising six brands. For each trial of the testing, yoked label sections were randomized positionally so that any effect of placement of the information on the screen was mitigated.

We also analyzed the time it took to select a product for trials (where a correct selection was made within 60 seconds). As with the previous analysis, trials involving our mock commercial labels (see Fig 3) were not included in this analysis of the data. Gender, age, ethnicity, education level, visual acuity, health literacy, color blindness, ethnicity, and native languages were included in the model as explanatory covariates. The data was log transformed to meet normality assumptions. Similar to the analysis for the previous variable, linear predictors in this model were the four design factors.

Age (p<0.0001) and ethnicity (p = 0.0084) were retained in the final model, based on their Type III p values (α = 0.05). Correct choice location, which was randomized in each trial by EPrime^®^ software, had a significant effect (p = 0.0005) on the dependent variable (time to make a correct choice), and, as such, was included as a random variable in the final model, based on its Type III p values (α = 0.05). The model was fitted using the Mixed procedure of SAS (Version 9.3, SAS Institute, Cary, NC). Estimated least square means (LSM) and corresponding 95% confidence intervals (LCL = Lower Confidence Limit and UCL = Upper Confidence Limit) are reported here in the original millisecond scale. Relevant pairwise comparisons were conducted, using Fisher’s LSD.

The design variables, grouping (p = 0.0104), symbol use (p<0.0001) and color coding (p<0.0001) all positively impacted participants time to correct selection of a product. Reported estimates have been back-transformed for the following comparisons. Participants took significantly less time to make a correct choice when the pieces of critical information were grouped (LSM = 4.2 s, LCL = 3.6 s, UCL = 4.9 s), when compared with those that were not (LSM = 4.4 s, LCL = 3.8 s, UCL = 5.1 s). Symbol use also positively impacted the time to correctly select a product (LSM = 3.8 s, LCL = 3.3, UCL = 4.4) compared to labels that did not contain symbols (LSM = 4.8 s, LCL = 4.2 s, UCL = 5.6 s). The introduction of color (see Fig 2) also decreased time to correct selection of products ((p<0.0001) (LSM = 3.9 s, LCL = 3.4 s, UCL = 4.5 s) vs (LSM = 4.7s, LCL = 4.1s, UCL = 5.5s). No interaction terms yielded evidence of significant differences.

### Optimal Label

Since grouping, symbol presence and color-coding the designs provided evidence of a significant benefit on the time to make a correct choice, and symbol use provided evidence of enhancing the choice made (α = 0.01), we considered trials with this treatment combination (grouped information with symbols and color) to be the “optimized label”. In a subsequent analysis, we compared the results from these trials to the data from trials involving the two labels we developed based on current, commercial labels (Fig 3 for a trial comprised of one of the two labels developed).

**Fig 4:**
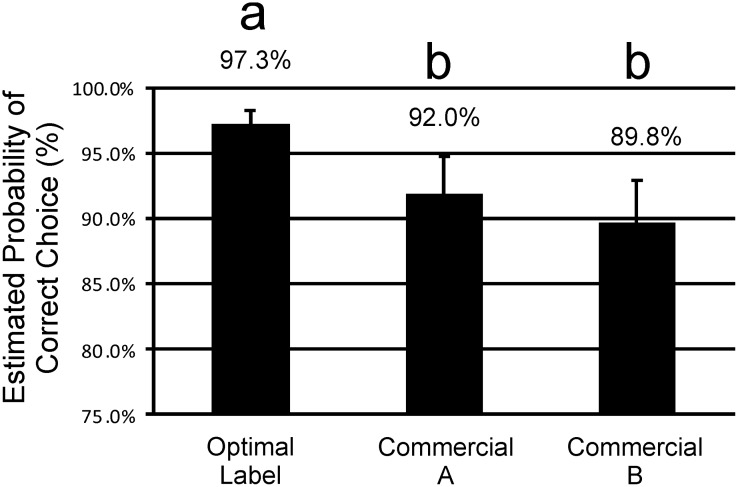
Probability of Correct Selection. Comparison of the optimized label and two designs based on current commercial practice for probability of correct choice means with upper and lower limits. Differing letters indicate statistical significance.

The probability of a correct choice of product was significantly greater for our optimized label design (97.3%, LCL = 95.5%, UCL = 98.4%), as compared with either of the commercial labels we tested (92.0%, LCL = 87.9%, UCL = 94.7%, and 89.8%, LCL = 85.3%, UCL = 93.0%). No evidence of a difference was apparent when the two commercial designs were compared (α = 0.01) (Fig 4).

**Fig 5:**
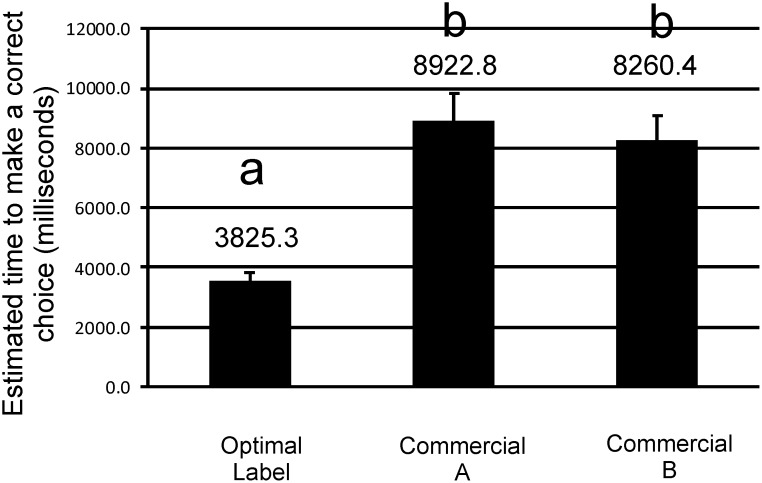
Estimated Time to Correct Selection. Comparison of the optimized label and two designs based on current commercial practice on time to make a correct choice: Estimated least square means (LSM) with estimated upper and lower limits. Differing letters indicate statistical significance.

The continuous variable, time to correctly select the product, also was positively impacted when our optimized label was compared with the two commercially based designs (α = 0.01) optimal design (LSM = 3.5 s, LCL = 3.2 s, UCL = 3.8 s), as compared to the two commercial labels we tested (LSM = 8.9 s, LCL = 8.1 s, UCL = 9.8 s and LSM = 8.2 s, LCL = 7.5 s, UCL = 9.1 s) (Fig 5).

## Discussion

Our objective was to evaluate the effect of four design factors (grouping, boxing, symbol use and color) when used with three pieces of critical information (latex status, sterility status and expiration date) to quantify their effect in an attempt to optimize label information. The performance of the optimized label was then compared with two labels based on standard, commercial practice to see if the solutions suggested by focus groups comprised of healthcare providers yielded improved performance (as indicated by the selection of correct products and time to correct selection).

The sample population for this study was composed of an experienced pool of healthcare providers. Further, by sampling at conferences of a national professional organization, it is not unreasonable to assume we were getting engaged providers from across the nation.

Our optimal label (which employed symbols, the use of color and grouped information critical to product selection) significantly increased the probability of the selection of a correct product and reduced the time to make a correct choice, as compared to either of the labels we developed based on current commercial practice. Specifically, participants responded correctly in trials testing the optimal labels in approximately half the time, compared to the two commercial labels.

## Conclusions

Our study provides the empirical evidence that is currently lacking in the small body of literature concerning the labeling of medical devices, and suggests improvements can be made that have the potential to significantly impact the ability to differentiate products and the time to select the same.

Data are available in a flat file labeled as S1 Table.

## Supporting Information

S1 Table Raw Data Flat File. (XLSX)

## Abbreviations

AST: Association of Surgical Technologists
BIRB: Biomedical and Health Institutional Review Board
CFR: Code of Federal Regulations
CI: Confidence Interval
EU: European Union
LCL: Lower Confidence Limit
LSM: Estimated Least Square Means
UCL: Upper Confidence Limit
US FDA: United States Food and Drug Administration
